# Spectral analysis for monitoring mangrove restoration: A case study in the Vietnamese Southern Coastline

**DOI:** 10.1017/cft.2025.5

**Published:** 2025-03-28

**Authors:** Thuong V. Tran, Ruth Reef, Xuan Zhu, Midhun Mohan

**Affiliations:** 1 School of Earth, Atmosphere and Environment, Monash University, Clayton, VIC, Australia; 2 Ecoresolve, San Francisco, CA, USA; 3 Department of Geography, University of California-Berkeley, Berkeley, CA, USA

**Keywords:** NDVI, Mann–Kendall significant, Hurst exponent, time series imagery, Landsat, blue carbon conservation

## Abstract

Mangrove restoration efforts have been ongoing, but with varying levels of success, requiring spatial and temporal monitoring to better understand the stocks and drivers of success. Here, we used multi-spectral remote sensing and spatial regression techniques to examine mangrove distribution and restoration potential in the Vietnamese Southern Coastal (VSC) region from 1988 to 2023, an area where multiple episodes of mangrove restoration have been attempted over the past decades. Our results show that 51.5% of the mangrove area has recovered from previous losses, while 48.5% has been lost during the 1988–2023 period. Significant gains were observed between 2018 and 2023, accounting for 77.8% of the total restoration. However, over 40,000 ha of mangroves were lost during each decade between 1988 and 2018, primarily due to land-use changes. Regression analyses estimated a sustainable mangrove cover increase of 9.9% (23,407 ha) and persistence of 22.5% (52,936 ha), mainly in protected areas and low-impact zones. Conversely, 9.8% (23,056 ha) of mangroves in erosion-prone and human-disturbed regions face continued decline. Our study demonstrated the effectiveness of integrating long-term Normalised Difference Vegetation Index time-series analysis with spatial regression to monitor mangrove ecosystems. These techniques offered a scalable framework for global mangrove monitoring and restoration planning, supporting evidence-based conservation policies.

## Graphical abstract

**Figure:**
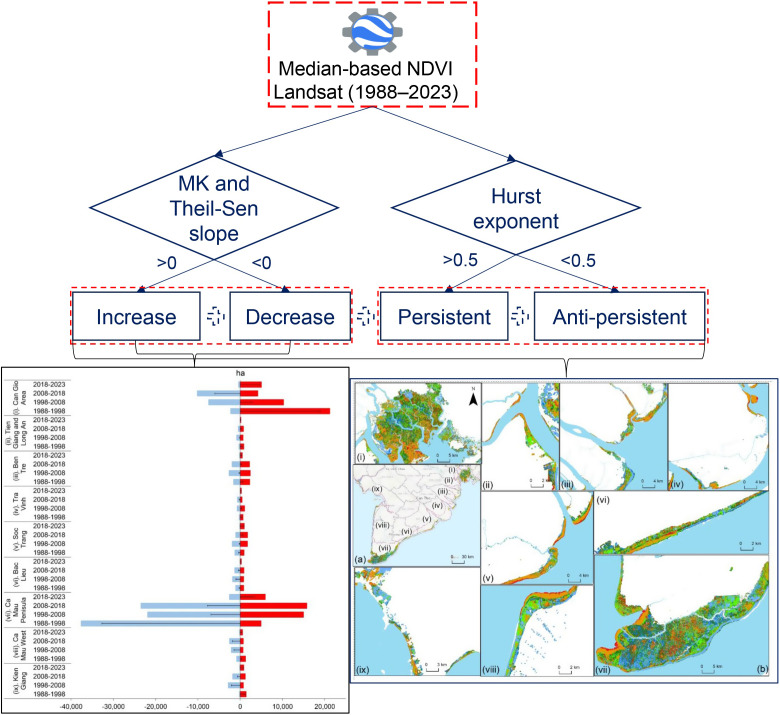

## Impact statement

Attempts to restore mangrove ecosystems have been gaining in popularity, but most attempts have been unsuccessful due to failure to include the wide range of factors influencing these systems. Restoring degraded ecosystems requires reliable, data-driven approaches to assess long-term changes before and after restoration, which can then guide conservation efforts. Remote sensing and spatial regression frameworks can enhance our ability to monitor the wide range of factors affecting mangroves and the effectiveness of mangrove restoration across wide scales. Here, we describe a transferrable approach that combines Theil-Sen slope analysis, Mann–Kendall trend testing, and Hurst exponent analysis to detect long-term vegetation dynamics and use this methodology to identify mangrove degradation hotspots and assess restoration feasibility in Vietnam. Our findings underscore the effectiveness of conservation policies in promoting natural regeneration and mitigating human-driven losses. By distinguishing between protected and unprotected mangrove areas, this research delivered actionable insights for policymakers, coastal managers and restoration practitioners, contributing to global efforts in ecosystem resilience, climate adaptation and disaster risk reduction.

## Introduction

Mangrove ecosystems are essential for coastal resilience, biodiversity conservation and climate change mitigation (Bunting et al., 2022). Their functions include offering natural barriers and protecting shorelines from erosion, storm surges and rising sea levels, while providing critical habitats for diverse marine and terrestrial species. Additionally, mangroves are highly effective carbon sinks, sequestering more carbon per unit area than most terrestrial forests, thus playing a crucial role in mitigating global climate change (Rondon et al., 2023). Despite their ecological importance, mangroves are rapidly degrading because of human activities such as deforestation, aquaculture expansion and urban development, especially in Southeast Asia (Goldberg et al., 2020; Gerona-Daga and Salmo, 2022). This degradation not only increases the vulnerability of coastal communities to environmental threats but also results in biodiversity loss and increased carbon emissions. As such, restoring mangrove ecosystems is imperative to enhance coastal protection, sustain biodiversity and achieve global climate objectives. In recent years, the management and conservation of mangrove ecosystems have received worldwide attention, and methods for the effective monitoring of mangroves have been proposed (Sam et al., 2023; Sunkur et al., 2023). However, the complexity of mangrove ecosystems, driven by their dynamic response to environmental changes, presents challenges for restoration efforts (López-Portillo et al., 2017; Gerona-Daga and Salmo, 2022). Effective restoration strategies require a thorough understanding of mangrove resilience, regeneration capacity and spatiotemporal dynamics (Ellison and Felson, 2020), thus developing adaptive, region-specific restoration approaches that can address both current degradation and future environmental challenges requires long-term datasets.

The increasing global use of remotely sensed data has transformed the ability to characterise mangrove dynamics, particularly through time series image datasets. Remote sensing has become an essential tool for monitoring and assessing mangrove ecosystems, offering a scalable and efficient alternative to traditional field-based methods, such as transects and plot-based measurements (Bunting et al., 2022; Rondon et al., 2023). While conventional approaches provide valuable insights at localised scales, they are limited in their ability to capture large-scale spatial heterogeneity and temporal variation. In contrast, remote sensing, particularly satellite imagery, enables comprehensive and continuous monitoring of mangrove ecosystems over extensive areas (Guo et al., 2021; Lu and Wang, 2021). Among various remote sensing indices, the Normalised Difference Vegetation Index (NDVI) has proven to be a highly effective indicator of vegetation health, growth and canopy density—key metrics for evaluating the success of mangrove restoration initiatives (Tran et al., 2022; Mohan et al., 2024). Landsat, with its 30-m spatial resolution and long-term (>35 year) data archive, provides a significant advantage for monitoring mangrove ecosystems over other sensors. For example, compared to higher-resolution sensors like Sentinel-2 (10-m resolution), Landsat provides an optimal balance between spatial coverage and temporal frequency, making it particularly well-suited for long-term assessments of mangrove dynamics (Tran et al., 2021). This temporal depth enables the detection of both short-term fluctuations and long-term trends in mangrove distribution and recovery, offering a more comprehensive understanding of habitat suitability and restoration trajectory.

Numerous change detection algorithms have been developed to analyse time-series remote sensing data in mangroves, particularly for detecting both gradual and abrupt changes in hydrological, vegetative and climatic factors (Militino et al., 2020; Meng et al., 2021; Tran et al., 2021). Previous research often employed singular approaches, such as using individual sensors or specific change detection algorithms, to assess mangrove ecosystems (Le et al., 2020; Tinh et al., 2022). For instance, linear trend analysis provides valuable information on vegetation dynamics, indicating whether mangrove cover in a given area is increasing or decreasing over time (Le et al., 2020). However, this approach is insufficient to assess the long-term sustainability of observed trends. The Hurst exponent complements this by examining whether detected changes follow a predictable, long-term pattern (persistence) or are just short-term fluctuations (anti-persistence) (Tong et al., 2018; Igbawua et al., 2019). Despite its strengths, the Hurst exponent can be sensitive to noisy data or shorter time series, which may affect its accuracy in certain contexts. To overcome these limitations, combining multiple methods offers a more robust and comprehensive framework to examine time series of change. Recent advances in mangrove monitoring have integrated linear trend analysis with the Hurst exponent to assess both the direction and sustainability of vegetation change, particularly in tropical and subtropical mangrove ecosystems (Zhu et al., 2021; Wen et al., 2023). This combination has proven to be a significant improvement over traditional methods, offering a more nuanced understanding of the temporal dynamics of mangrove restoration.

In this study, we applied an integrated approach to provide a comprehensive understanding of mangrove restoration efforts over the past few decades. By combining trend detection with long-term sustainability analysis, we aimed to generate a detailed spatiotemporal assessment of mangrove distribution and regeneration trends between 1988 and 2023 and to explore the potential regions of mangrove restoration through spatial regression and remote sensing analysis. The Vietnamese Southern Coastline was chosen as the case study area due to its long history of mangrove degradation and restoration efforts. The region has undergone significant ecological transformations over the past century, from widespread deforestation during the Vietnam War and post-war economic development era to modern large-scale restoration programs in recent decades (Nam and Sinh, 2014; Tinh et al., 2022; Tran et al., 2024a). By examining the trends and sustainability of mangrove recovery, the research not only supports Vietnam’s ongoing mangrove conservation strategies but also provides a model for other coastal regions facing similar environmental challenges.

## Site description

**Figure 1.**
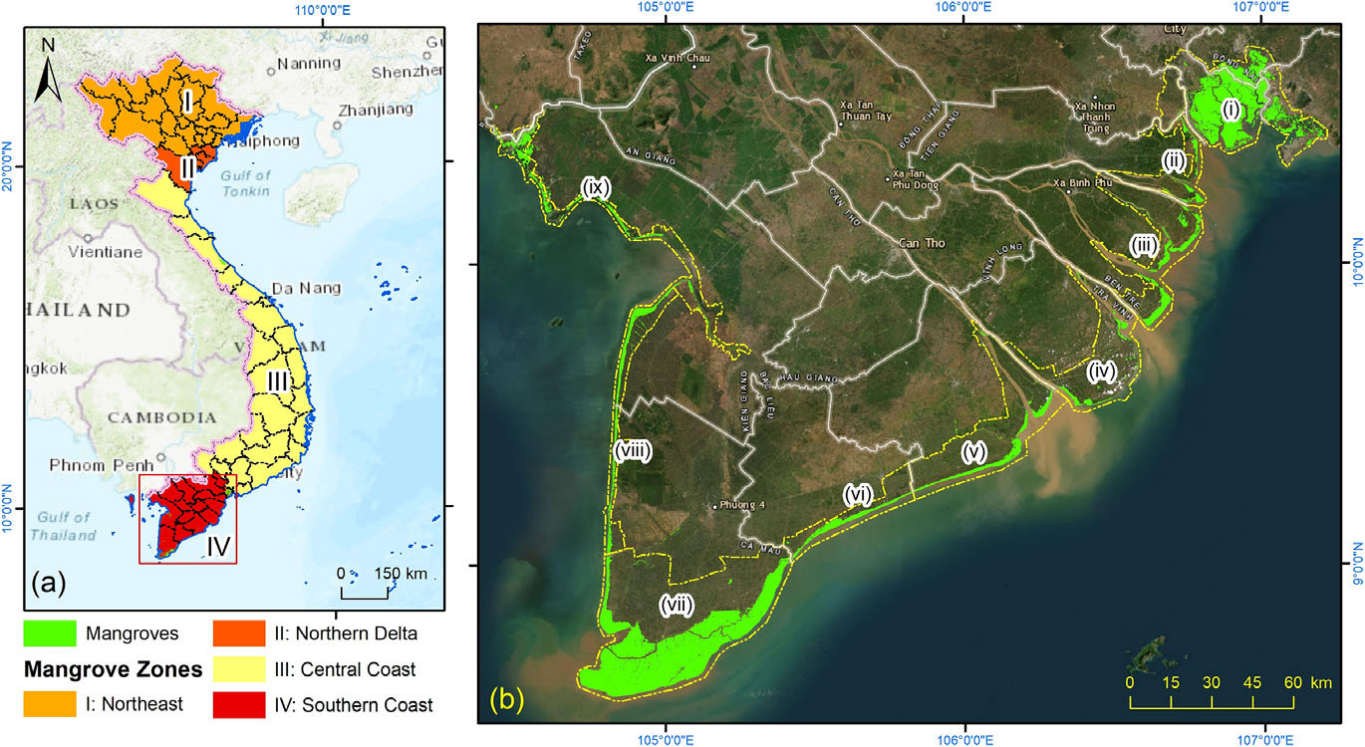
(a) Map showing the location of the study area and the four mangrove zones within Vietnam. (b) Total mangrove distribution (highlighted in light green) within the VSC region during the 1996–2023 period. This map is adapted from data provided by the Global Mangrove Watch (Giri et al., 2011), published VSC mangrove products (Tran et al., 2024b) and supplemented with local insights. The numbered list is formatted from (i) to (ix) as follows: (i) Can Gio area, (ii) Long An, (iii) Tien Giang, (iv) Ben Tre, (v) Tra Vinh, (vi) Soc Trang and Bac Lieu, (vii) Ca Mau, (viii) Ca Mau West and (ix) Kien Giang. This numbering scheme will be consistently applied in the subsequent figures presented in Section “Results.”

The southern coast of Vietnam or Vietnamese Southern Coastline (VSC), located between latitudes 8°34′00″–10°42′42″ N and longitudes 104°28’55″–107°04′59″ E, extends from Vung Tau Cape (Ba Ria–Vung Tau) in the east to Ha Tien (Kien Giang) in the west. The region includes the extensive mangrove ecosystems of the Mekong River Delta and the Can Gio Biosphere Reserve along the eastern shorelines of south Vietnam, covering an area exceeding 100 km^2^ (Figure 1). Dominant mangrove genera, such as *Rhizophora* and *Avicennia*, are key components of natural defence systems against coastal hazards, including landslides, erosion and rising sea levels.

Historically, the region has experienced severe deforestation due to war, timber and agricultural expansion, which has drastically reduced mangrove coverage (Tinh et al., 2022; Tran et al., 2024b). The Vietnam War had a particularly devastating impact, with more than 150,000 ha of mangroves damaged by bombing and chemical defoliants. Post-war economic challenges further accelerated mangrove loss, especially between 1976 and 1990, as land use shifted to accommodate agriculture and aquaculture. However, restoration efforts began in the late 1970s and have since gained momentum (Nam and Sinh, 2014), resulting in a mosaic of deforestation, natural forests and restored sites of varying ages.

Various national and international programs have focused on the rehabilitation of these ecosystems, with restoration projects aimed at reversing mangrove loss through reforestation and protection policies (Pham et al., 2022). A significant government replanting project in 1992 restored 52,000 ha, positioning the VSC as a leading example of large-scale mangrove restoration. This success has been internationally recognised, with areas such as Can Gio and the Ca Mau Peninsula designated as UNESCO Biosphere Reserves (Tran et al., 2024b). Despite these efforts, ongoing pressures from aquaculture, urbanisation and timber exploitation continue to threaten the long-term sustainability of the region’s mangroves. Urban growth and the expansion of shrimp farming, particularly in the Mekong Delta, have contributed to the fragmentation and degradation of mangrove forests (Pham et al., 2022; Phan and Stive, 2022; Tinh et al., 2022). This makes the VSC an essential focus for research on mangrove restoration and provides insights into the challenges and successes of conservation under multiple human-induced stressors. Given the vulnerability of mangrove ecosystems and the increasing focus on their restoration, it is crucial to understand the patterns of mangrove growth in order to locate suitable areas for regeneration activities and optimise survival rates.

## Material and methods

### Earth observation data

Landsat products, including Landsat 5 TM, Landsat 7 ETM+, Landsat 8 and Landsat 9 OLI/TIRS, with a spatial resolution of 30 m, were acquired from Google Earth Engine (GEE, https://earthengine.google.com/) for the 1988–2023 period (Gorelick et al., 2017). To monitor mangrove distribution, we utilised the median annual NDVI, calculated as (Red – Near Infrared) / (Red + Near Infrared) (Rouse et al., 1973). NDVI is a widely used remote sensing metric, particularly effective for assessing mangrove health and density, making it ideal for detecting changes in mangrove cover (Tran et al., 2022). Image filtering and pre-processing were conducted through GEE. In addition to satellite data, we incorporated Global Mangrove Watch data for the 1996–2020 period (Bunting et al., 2022) and time-series datasets of mangroves along the southern coast of Vietnam (Tran et al., 2024b) to establish the mangrove boundary for the study area. By integrating these datasets, we enhanced the accuracy of mangrove mapping by incorporating local knowledge to address potential misclassifications in global datasets, ensuring more precise differentiation between mangrove and terrestrial forests. A 3-km buffer was applied to ensure that the analysed area is sufficiently large to minimise classification errors caused by mixed pixels in Landsat imagery and to accurately delineate areas corresponding to mangrove distribution for further analysis.

### Changes in mangrove distribution

To detect long-term trends in mangrove restoration, two widely recognised statistical methods were employed: Theil-Sen median trend analysis and the Mann–Kendall test (Mann, 1945; Sen, 1968). These methods are invaluable for environmental research due to their robustness against outliers and non-normal data distributions (Zhu et al., 2021). These methods allowed us to quantify the rate of change in mangrove cover (through NDVI) and to determine where significant trends occurred. The NDVI values with a threshold range of ±1 from 1988 to 2023 were used to compute the trend for each pixel using Theil-Sen slope, capturing the temporal changes in the mangrove distribution.

Given a time series of NDVI values 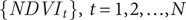 , where 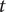 represents the year and 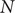 represents the number of years (36 years), the slope between 2 years 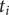 and 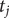 is calculated as follows in Equation (1):
(1)

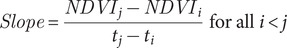



The Theil-Sen estimator is the median of all slopes as shown in Equation (2):
(2)

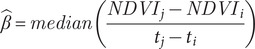



By calculating this slope at each pixel, we obtained a spatial representation of the rate of change in mangroves. Positive values of 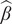 indicate increasing NDVI, suggesting mangrove restoration, while negative values suggest mangrove ongoing degradation.

Afterwards, we applied the Mann–Kendall test to assess the statistical significance of the trends detected by the Theil-Sen analysis. This test evaluates whether there is a monotonic upward or downward trend in NDVI over the 1988–2023 period at each pixel. The test is based on comparing each NDVI value with every subsequent value to determine if there is a consistent increase or decrease. For a time-series of NDVI values 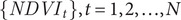 , the Mann–Kendall test statistic 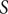 is calculated using Equation (3–4) as follows:
(3)

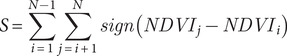



where the sign function is defined as follows:
(4)

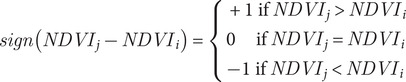



Mann–Kendall test results identified statistically significant trends, confirming whether the observed changes were positive (indicating restoration/increase) or negative (indicating degradation/decrease). A *p-*value of less than 0.05 was used to determine the statistical significance of the trends, with values below this threshold indicating that the trends are statistically significant. Based on the Theil-Sen slope and Mann–Kendall test results presented in Section “Results,” the spatiotemporal trends of the mangrove forests were classified into five distinct categories, as outlined in Table 1. The classified levels reflect both increasing and decreasing trends, encompassing natural and managed restoration or reduction processes. Natural restoration refers to the autonomous regeneration of mangroves, often leading to more resilient and biodiverse ecosystems. Managed restoration involves human interventions, such as planting and hydrological adjustments, to facilitate recovery, especially in areas where natural regeneration is impeded by factors like altered sediment flow or disrupted hydrology. Conversely, reductions in mangrove cover can result from natural events or human activities, including deforestation and coastal development. This classification method, derived from a combination of these two statistical approaches, has been widely applied in several studies on mangrove remote sensing (Le et al., 2020; Zhu et al., 2021).

**Table 1.** Categories of trend in mangrove distribution using Theil-Sen estimator (slope) and Mann–Kendall test (Z-score)

Slope	Z-score	Trend level
≥0.0005	≥1.96	Significant restoration/increase
≥0.0005	−1.96 to 1.96	Slight restoration/increase
−0.0005 to 0.0005	−1.96 to 1.96	Stability
<−0.0005	−1.96 to 1.96	Slight reduction
<−0.0005	<−1.96	Significant reduction

### Long-term persistence trends of mangrove distribution

In the context of mangrove restoration, we applied the Hurst exponent to assess the long-term behaviour of the mangrove ecosystem, specifically its tendency towards sustained growth or decline. By calculating the Hurst exponent for each pixel, we measured the predictability of changes in mangrove cover, categorising regions based on their temporal dynamics (Tong et al., 2018; Tran et al., 2021). A value of H = 0.5 indicates random fluctuations, suggesting natural variability without a clear pattern of restoration or degradation. Values between 0.5 and 1 represent regions with persistent trends, where past increases in NDVI are likely to continue, indicating ongoing restoration. Conversely, values between 0 and 0.5 suggest anti-persistent or mean-reverting behaviour, where past increases in mangrove cover are likely to be followed by decreases, highlighting instability or degradation. In this study, the Hurst method was implemented to systematically evaluate the temporal dynamics of mangrove restoration. A stepped breakdown of the process and its corresponding formula is provided below:

(i) Define the time series: Let the time series 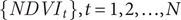 represent the annual NDVI values for each pixel over the 36-year period.

(ii) Compute the mean sequence: For each pixel, compute the mean *μ* of the NDVI time series using Equation (5):
(5)

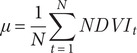



(iii) Calculate the accumulated deviation: This step transforms the NDVI series into an accumulated sum centred around the mean, allowing us to evaluate the fluctuations from the average behaviour. The accumulated deviation 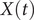 from the mean is calculated as Equation (6):
(6)

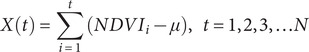



(iv) Create the range sequence: For each time step, compute the range between the maximum and minimum accumulated deviations. The range sequence 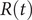 over time the period *t* is defined as Equation (7):
(7)

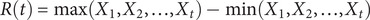



(v) Obtain the standard deviation sequence: The standard deviation 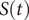 quantifies the variability of the NDVI values over time and is obtained as Equation (8):
(8)

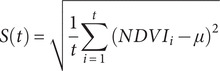



(vi) Calculate the rescaled range: The rescaled range 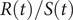 is the ratio of the range to the standard deviation. This metric captures the magnitude of fluctuations relative to the variability of the series.

(vii) Finally, the Hurst exponent is obtained by applying a log–log regression to the rescaled range 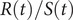 and time 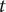 as Equation (9):
(9)

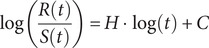



where 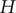 is the slope of the linear regression and represents the Hurst exponent and 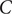 is a constant, typically 1.

## Results

### Changes in mangrove distribution between 1988 and 2023

The VSC region experienced a degradation of 68,842 ha (−48.5%) and a restoration of 73,117 ha (51.5%) between 1988 and 2023, resulting in a positive net change of 4,275 ha (+3.0%), demonstrating the success of recent restoration efforts (Table 2). The period from 1988 to 2018 saw significant mangrove degradation, with losses of 45,980 ha (−33.6%) in the decade between 1988 and 1998, 42,783 ha (−31.2%) in the decade between 1998 and 2008 and 43,958 ha (−32.1%) in the decade 2008–2018. However, restoration efforts during these periods mitigated some of the losses, with 34,841 ha (31.0%) restored from 1988 to 1998, 33,860 ha (30.2%) from 1998 to 2008 and 28,465 ha (25.4%) from 2008 to 2018. Despite these efforts, net losses were recorded for each of these decades. The trend shifted in the most recent period (2018–2023), where degradation dropped significantly to just 4,301 ha (−3.1%), while restoration surged to 15,100 ha (13.5%), resulting in a net gain of 10,799 ha (+10.4%).

**Table 2.** Decadal change (ha) in mangrove area in the VSC from 1988 to 2023

	1988–1998	1998–2008	2008–2018	2018–2023	1988–2023
Degradation/decrease	−45,980 (−33.6%)	−42,783 (−31.2%)	−43,958 (−32.1%)	−4,301 (−3.1%)	−68,842 (−48.5%)
Restoration/increase	34,841 (31.0%)	33,860 (30.2%)	28,465 (25.4%)	15,100 (13.5%)	73,117 (51.5%)
Net change	−11,139 (−2.6%)	−8,923 (−1.0%)	−15,493 (−6.7%)	10,799 (+10.4%)	4,275 (+3.0%)

**Figure 2.**
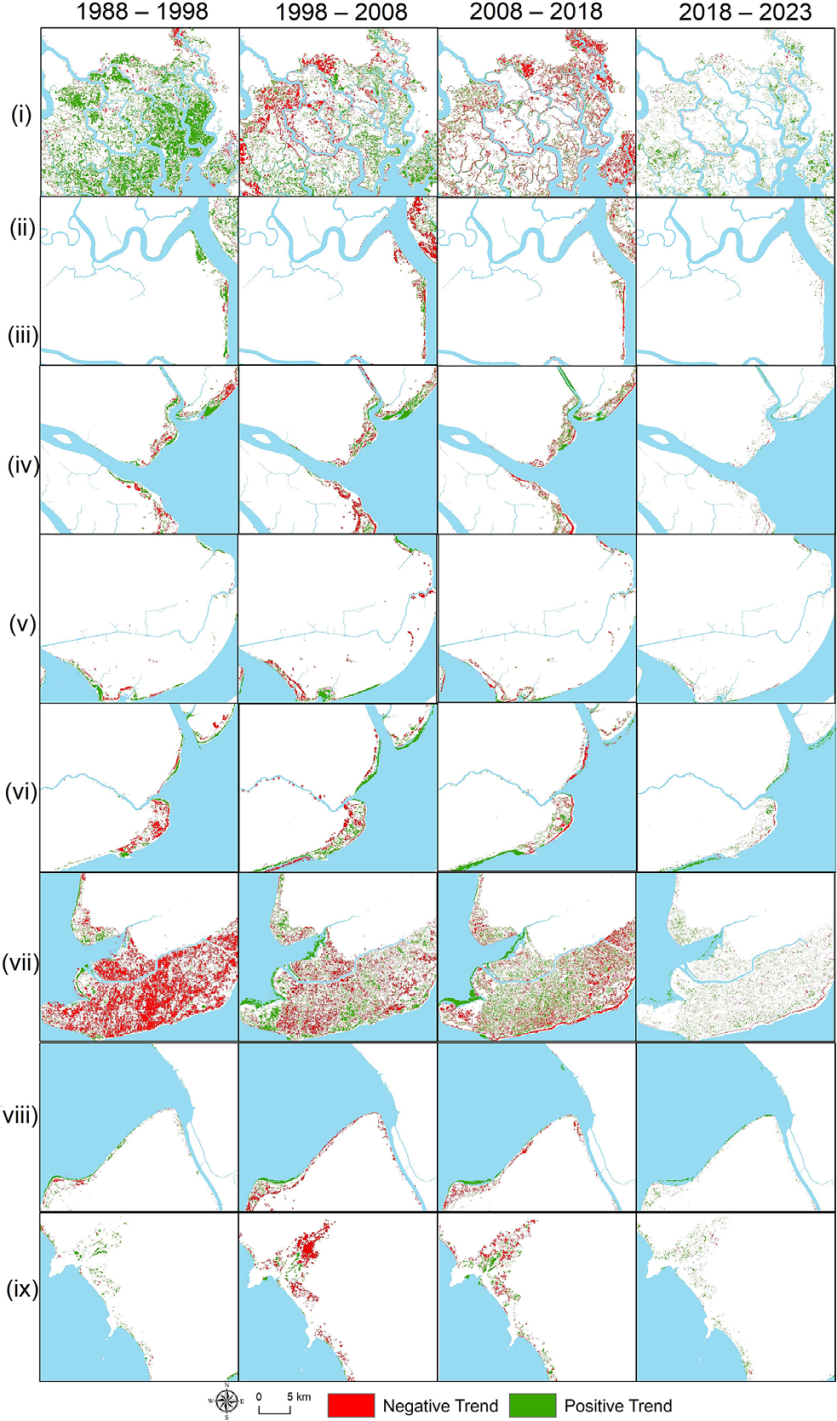
A comprehensive visualisation of the spatial and temporal trends in mangrove areas across the Vietnamese Southern Coastal (VSC) region, segmented into four key periods: 1988–1998, 1998–2008, 2008–2018 and 2018–2023. The numbered list is formatted as follows: (i) Can Gio area, (ii) Long An, (iii) Tien Giang, (iv) Ben Tre, (v) Tra Vinh, (vi) Soc Trang and Bac Lieu, (vii) Ca Mau, (viii) Ca Mau west and (ix) Kien Giang.

Regarding the changes in mangrove distribution through space and time, Can Gio area (Figure 2.i) showed significant restoration in the earlier period (1988–1998) with some declines in the following decades. In contrast, regions like the Ca Mau Peninsula (Figure 2.vi) and western regions (Figure 2.vii) exhibited widespread degradation, especially between 1998 and 2018, with some recovery during the 2018–2023 period. Other areas, such as Ben Tre (Figure 2.iii) and Soc Trang and Bac Lieu (Figure 2.v), reflected similar patterns of mangrove decline with intermittent recovery phases. However, in the most recent period (2018–2023), there was a notable recovery, with many areas turning green, signifying a positive trend in mangrove restoration.

**Figure 3.**
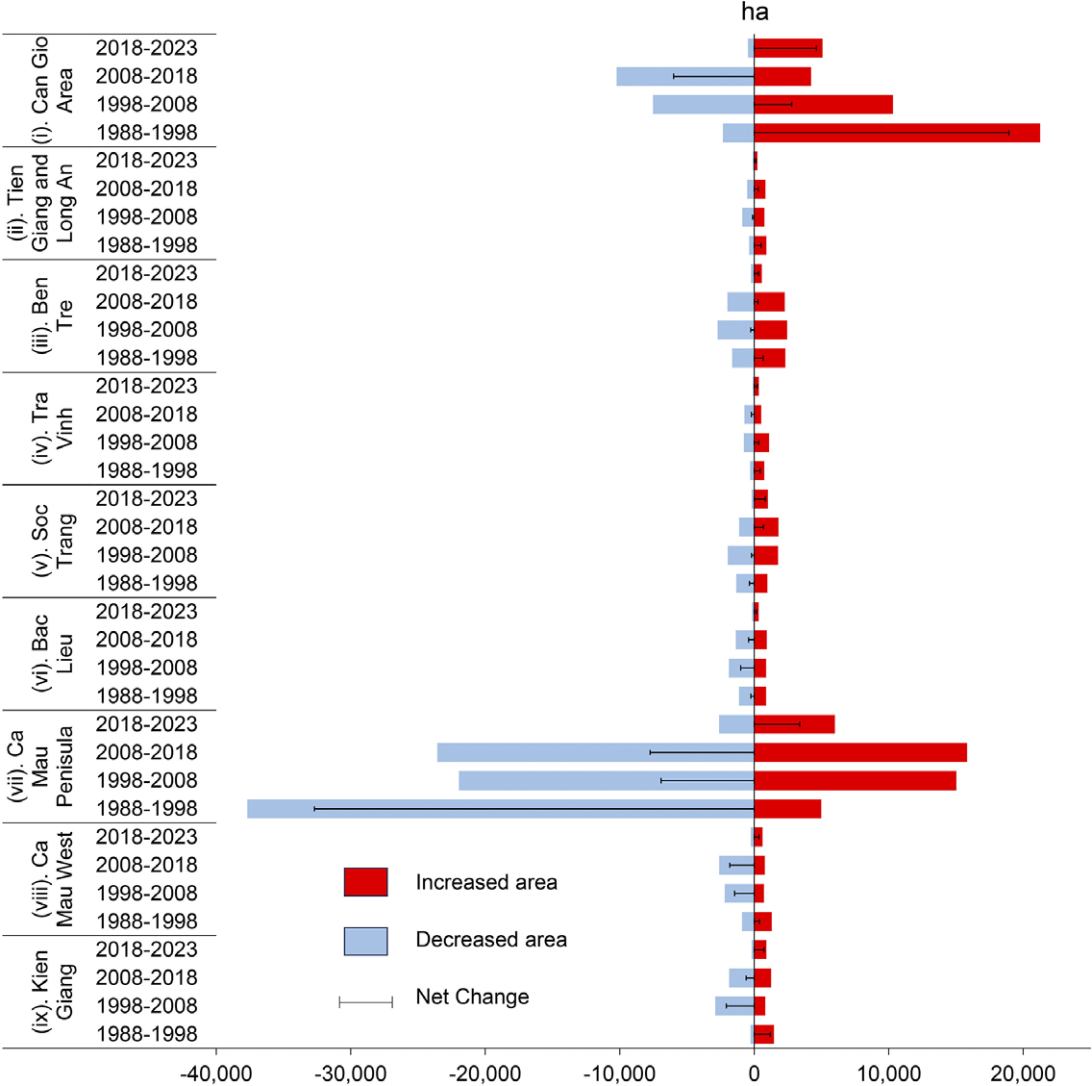
Mangrove cover change patterns in various regions of the southern Vietnamese coast from 1988 to 2023, segmented into four periods: 1988–1998, 1998–2008, 2008–2018 and 2018–2023. The trends observed in this figure correspond to the NDVI Sen’s slope analysis presented in Figure 2 (*p < 0.05*). An increase in the area reflects a greater extent of mangrove restoration.

The most significant restoration efforts occurred in Can Gio, Soc Trang and Tra Vinh, particularly in recent years, while Ca Mau Peninsula, Ca Mau West and Kien Giang suffered the greatest long-term degradation (see Figure 3). The Can Gio area stood out as the region with the most successful restoration, especially between 1988 and 1998, when it experienced a 90.2% gain in mangrove cover, and again in 2018–2023 with an 83.4% increase, resulting in an overall net gain of 24.4% over the entire period. Tra Vinh and Soc Trang also saw significant restoration, with net increases of 56% and 49%, respectively, driven by large gains in the 2018–2023 period, where Soc Trang saw an 84.3% gain and Tra Vinh experienced a 77.7% gain. On the other hand, regions like Ca Mau Peninsula, Ca Mau West and Kien Giang showed substantial degradation. The Ca Mau Peninsula was the most severely affected, with an 88.3% loss between 1988 and 1998 and continued degradation until 2018, before recovering slightly with a 69.6% gain between 2018 and 2023. Ca Mau West also faced heavy degradation, losing 72.2% of its mangrove cover between 1988 and 2023, although it saw some recovery in the last period (2018–2023) with a 70.9% gain. Kien Giang similarly experienced major degradation, particularly between 1998 and 2008, where a 77.9% loss occurred, and by 2023, it had an overall net loss of 32.7%. Ben Tre and Bac Lieu showed mixed trends, with early losses followed by moderate gains in later periods, resulting in modest net increases of 14.3% and 18.5%, respectively.

These results align with decadal trends in the conversion between mangrove and non-mangrove areas. In 1988, mangroves dominated the landscape with 222,461 ha, while non-mangrove areas were minimal. Over the next decade, the total mangrove area slightly increased to 223,642 ha in 1998, reflecting ongoing conservation and restoration efforts. However, during this period, some mangrove areas transitioned to non-mangroves, as indicated by the red flows from mangroves to non-mangroves, suggesting possible deforestation or land-use change for development purposes. Similarly, the diagram shows the persistence of a majority of the mangrove cover but also highlights shifts in land cover between classes. From 1998 to 2008, a minor decline in mangrove areas occurred, dropping to 222,626 ha, while non-mangrove areas slightly increased. This trend remained relatively stable through the 2018 period, where mangrove areas reached 223,539 ha. By 2023, mangrove areas totalled 222,727 ha, showing remarkable resilience despite pressures from coastal development and climate change. Non-mangrove areas consistently increased, but the large green flows indicate that most mangrove areas have been preserved or restored over the years.

### The consistent trend of mangrove restoration in the study area

The sustainability of mangrove forests was analysed using the Hurst exponent alongside Theil-Sen median trend analysis and the Mann–Kendall test at a significance level of 95%, categorising results into six levels of sustainability based on the persistence and change in NDVI over time. The findings revealed a diverse spatial distribution of sustainability trends in mangrove forests (Figure 5) and their areas (ha) in the 1988–2023 period (Table 3). Areas experiencing anti-persistence and significant loss—indicating non-sustainability with continued degradation—covered 45,214 ha, accounting for 19.2% of the total area. In contrast, areas with persistence and significant loss, representing long-term degradation, totalled 23,056 ha or 9.8% of the region. On the other hand, regions with anti-persistence and stability, where changes were non-sustainable but stable, spanned 41,549 ha (17.7%). Persistence and stability, signalling long-term sustainable stability, covered the largest area, 52,936 ha (22.5%). Mangrove regions showing anti-persistence and significant gain—indicating rapid but unsustainable improvement—covered 49,161 ha (20.9%). Finally, areas with persistence and significant gain, where sustainable and long-term improvement was present, represented 23,407 ha or 9.9% of the total area. These results suggest a complex mix of sustainability dynamics, with nearly half of the mangrove regions experiencing some form of stability or improvement, while others are either degrading or unsustainable despite improvement efforts.

**Table 3.** Sustainability analysis

Categories	Area (ha)
Anti-persistence and significant loss	45,214 (19.2%)
Anti-persistence and stability	41,549 (17.7%)
Anti-persistence and significant gain	49,161 (20.9%)
Persistence and significant loss	23,056 (9.8%)
Persistence and stability	52,936 (22.5%)
Persistence and significant gain	23,407 (9.9%)

*Note*: Spatial distribution in terms of NDVI significant change.

## Discussion

### Drivers of mangrove changes in the VSC

**Figure 4.**
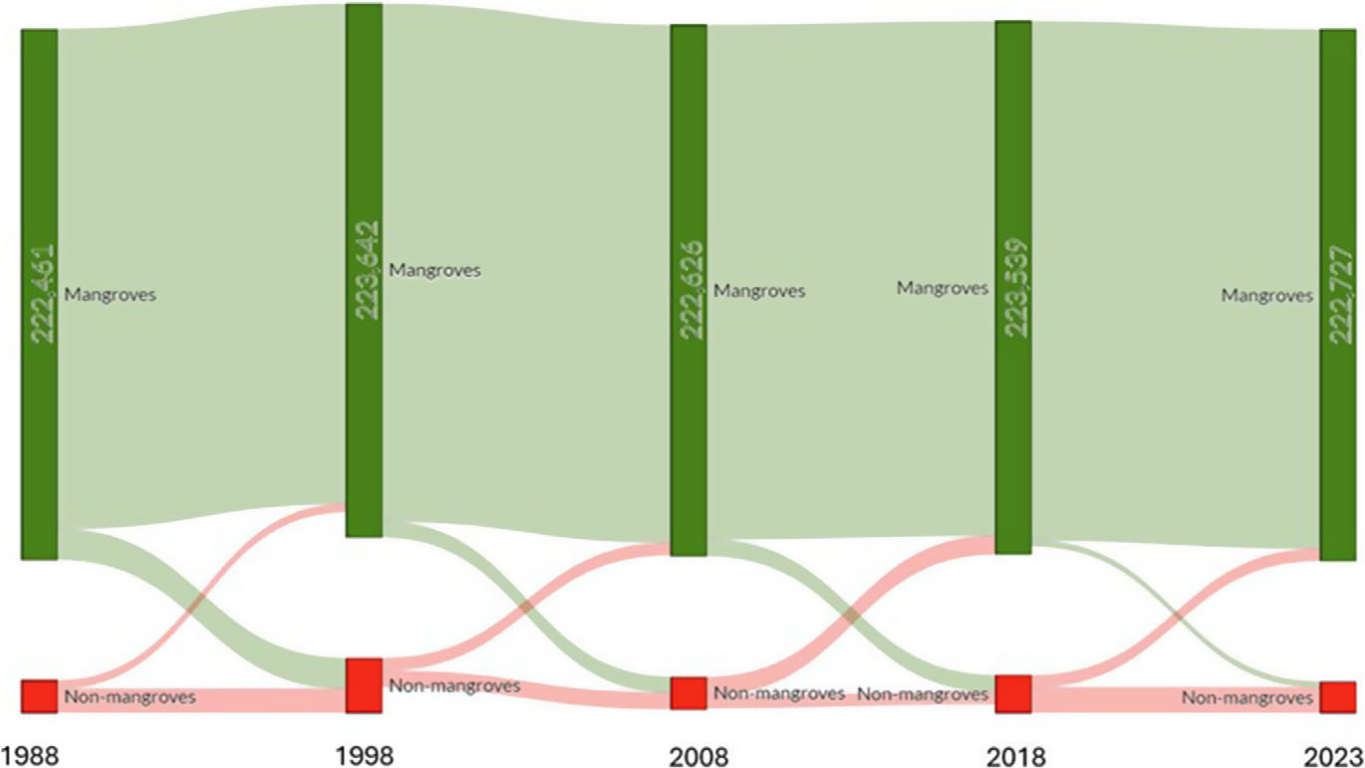
The Sankey diagram of mangrove and non-mangrove transition in the distributed mangroves of the VSC from 1988 to 2023. Each bar represents the total area (ha) classified as either mangrove (green) or non-mangrove (red) at five key intervals: 1988, 1998, 2008, 2018 and 2023. The widths of the green and red bars correspond to the total area covered by mangroves and non-mangroves, respectively, with values displayed horizontally along each bar.

NDVI values derived from multi-temporal Landsat imagery, combined with spatial regression analysis, were utilised to evaluate the spatial and temporal dynamics of mangrove ecosystems in the Vietnamese Southern Coastal (VSC) region from 1988 to 2023. Several distinct phases of mangrove ecosystem change were identified due to land-use land cover changes (Figure 4). The period from 1988 to 1998 marked significant degradation, primarily driven by the rapid expansion of aquaculture and urbanisation along the coastal areas (Tran et al., 2024a, 2024b). Degradation persisted between 2008 and 2018, further exacerbated by human activities and natural factors, including coastal erosion and extreme weather events. However, a recovery phase emerged between 2018 and 2023, supported by natural regeneration and intensified human interventions, such as government-led reforestation programs and improved protective measures. For instance, a newly established mangrove islet in the Ca Mau area, covering approximately 50 ha, emerged as part of a restoration program initiated by the Forest Department and the World Wildlife Fund in 2019 (Figure 6).

The transitional period between 1998 and 2008 presented both degradation and recovery, where some areas exhibited improvement due to habitat restoration, while others continued to experience persistent declines. The spatial distribution of degradation varied across the region, with marginal mangroves—more vulnerable to coastal erosion and human encroachment—experiencing significant losses compared to more sheltered, interior forests. Natural processes, such as erosion, impacted marginal areas, yet these regions demonstrated potential for regrowth when environmental conditions were favourable (Phan and Stive, 2022; Tinh et al., 2022). Conversely, anthropogenic pressures, including aquaculture expansion and infrastructure development, caused more permanent degradation, resulting in unsustainable conditions and often irreversible changes to land cover (Le et al., 2020; Tran et al., 2024b). Recent conservation and reforestation efforts, particularly from 2018 onwards, have led to notable gains in mangrove coverage, demonstrating the positive impact of targeted restoration programs (Tran et al., 2024a). These findings highlight the importance of continued monitoring and strategic intervention to ensure the recovery and long-term sustainability of mangrove ecosystems.

### Comparisons with other studies

Previous studies in the VSC predominantly employed dual-image methods to assess mangrove loss and gain (Pham et al., 2022; Phan and Stive, 2022; Tinh et al., 2022), while only a few utilised spatial regressions to examine changes in mangrove distribution (Le et al., 2020; Tran et al., 2024a). These studies largely attributed mangrove loss to land-use changes, particularly the expansion of aquaculture between 2010 and 2019. Our study aligns with these findings, particularly in regions like the Ca Mau Peninsula, where significant degradation occurred during the 1988–1998 and 2008–2018 periods (Figure 2.vii and Figure 3). However, the spatial regression analysis revealed a reversal of this trend from 2018 to 2023, showing a modest yet crucial net gain in mangrove cover, illustrating the positive impact of recent restoration efforts (Figure 6).

Additionally, previous research has often examined the Can Gio area and the Mekong River Delta (MRD) separately. However, these studies consistently highlighted the importance of promoting natural regeneration through both global and local mangrove planting initiatives, alongside policies aimed at improving water quality and addressing environmental concerns for the benefit of human well-being (Pham et al., 2022; Tinh et al., 2022). Our findings corroborated these approaches, particularly in regions such as Soc Trang (Figure 2.v) and the Ca Mau Peninsula (Figure 2.vii), where natural regeneration has contributed to the restoration’s success. However, slower recovery in areas like Ben Tre (Figure 2.iv) and Bac Lieu (Figure 2.vi) suggested the need for targeted interventions, such as hydrological improvements or managed realignment, to enhance restoration outcomes. The variability in restoration success across the VSC highlights the importance of tailored strategies that address local environmental factors and the extent of human disturbances.

In addition to comparing outcomes with previous studies, our research employed an integrated spatiotemporal analysis to unravel the complexity of mangrove ecosystem dynamics. By using a multi-method framework, we went beyond conventional trend analysis to evaluate both the direction and sustainability of mangrove changes from 1988 to 2023. The application of the Theil-Sen slope for robust trend estimation, combined with spatial regression, offered a nuanced view of how mangrove cover fluctuated over time and across regions. The incorporation of the Hurst exponent added another layer of insight, revealing the persistence and long-term sustainability of these changes, which is crucial for informing future restoration initiatives. This comprehensive approach not only tracked the trajectory of mangrove changes but also provided guidance on areas where recovery might be unsustainable.

The integration of these methods proved vital for assessing the effectiveness of recent restoration efforts across the VSC. The analysis of changes at multiple spatial scales—ranging from coastal to inland regions—allowed for a deeper understanding of the influence of natural and human factors on mangrove recovery. While reforestation projects were successful in specific coastal areas, the findings also highlighted regions where continued intervention is necessary to halt degradation. By combining trend analysis with sustainability assessments, our approach offers a robust tool for managing mangrove ecosystems, while balancing immediate restoration needs with long-term ecological resilience. This strategic framework can support adaptive management and better conservation outcomes, ensuring more efficient resource allocation and targeted conservation actions.

### The research contribution towards achieving the sustainable development goals

By analysing mangrove dynamics in the VSC region from 1988 to 2023 using multi-temporal Landsat data and spatial regression techniques, our study highlighted the critical role of mangrove ecosystems in sustainable coastal management and urban planning. The findings supported decision-making for SDG 11 (Sustainable Cities and Communities), SDG 13 (Climate Action) and SDG 15 (Life on Land) by providing evidence and insights into mangrove resilience, restoration potential and long-term sustainability.

Mangroves, as vital green spaces in coastal regions, align with SDG 11 by supporting the sustainability of urban environments. These ecosystems not only improve environmental health by reducing air pollution and sequestering carbon, but they also enhance urban resilience to natural disasters (Ferreira et al., 2022). Incorporating mangrove cover into coastal urban planning allows cities to mitigate the effects of climate change, creating spaces that are ecologically rich and beneficial for human well-being. This aligns with SDG targets 11.6 (reducing the environmental impact of cities) and 11.7 (providing safe, inclusive and accessible green spaces). With the Mekong River Delta (MRD) being highly vulnerable to the impacts of global warming, particularly rising sea levels, mangroves serve as natural defences, protecting coastal communities from erosion and extreme weather events (Schmitt et al., 2023). By generating accurate long-term data on mangrove cover trends, the study offers valuable insights needed for adaptive management strategies that improve both ecological resilience and human safety, supporting SDG 13’s focus on climate adaptation and disaster risk reduction.

Moreover, this research advanced SDG 15 by emphasising the conservation and restoration of critical ecosystems. Mangroves are biodiversity hotspots that provide essential ecosystem services, benefiting both human and environmental health (Spencer et al., 2016). The findings underscore the importance of restoring and preserving mangrove forests not only to combat biodiversity loss but also to strengthen natural carbon sinks, which are crucial for mitigating climate change.

Integrating mangrove monitoring into coastal management and urban development plans is vital for fostering sustainability across multiple levels. The study highlighted that protecting and restoring mangrove forests is not just a local concern but a global necessity, given their role in mitigating climate change, supporting biodiversity and contributing to resilient urban communities. The insights gained offer a strong foundation for future restoration initiatives, demonstrating that sustainable mangrove management is essential for achieving long-term ecological and social resilience. By addressing both natural and human-driven factors influencing mangrove changes, the research provided a pathway for informed decision-making, ensuring that local conservation efforts align with global sustainability goals.

### Considerations while undertaking mangrove restoration

Mangrove restoration in the VSC involves navigating a complex array of ecological and socio-economic challenges. Our study revealed positive restoration trends but underscores the importance of addressing key considerations before embarking on large-scale restoration or afforestation initiatives. Although artificial restoration techniques, such as planting, have been successful in specific areas, encouraging natural regeneration may present more sustainable long-term outcomes. Improving hydrological conditions is one such method, creating a more favourable environment for the natural regrowth of mangroves. In historically degraded regions such as Bac Lieu (Figure 2.vi) and Ca Mau (Figure 2.vii and viii), focusing on hydrological interventions may yield better results than extensive artificial planting. This approach promotes healthier ecosystems by allowing local species to re-establish themselves without the risk of monoculture, which can undermine biodiversity.

Engaging local communities and stakeholders is crucial for the enduring success of mangrove restoration (Chamberland-Fontaine et al., 2022; Sathiyamoorthy and Sakurai, 2024). Projects that involve local populations have a higher likelihood of success, as these communities are directly impacted and have a vested interest in the outcomes. Examples of mangrove restoration activities in the study area, demonstrating the collaboration between government bodies, local residents and other stakeholders are shown in Figure 7. In areas such as Can Gio (Figure 2.i) and Ca Mau (Figure 2.vii), where both degradation and restoration trends have been observed, involving residents in the planning and execution phases fosters a sense of ownership over restored ecosystems. This involvement also opens pathways for alternative livelihoods, including eco-tourism and sustainable aquaculture, reducing pressures from harmful activities like illegal logging and intensive shrimp farming. Educating these communities about the ecological and economic benefits of mangroves can further enhance the long-term protection of restored areas, thus reinforcing the success of conservation initiatives.

**Figure 5.**
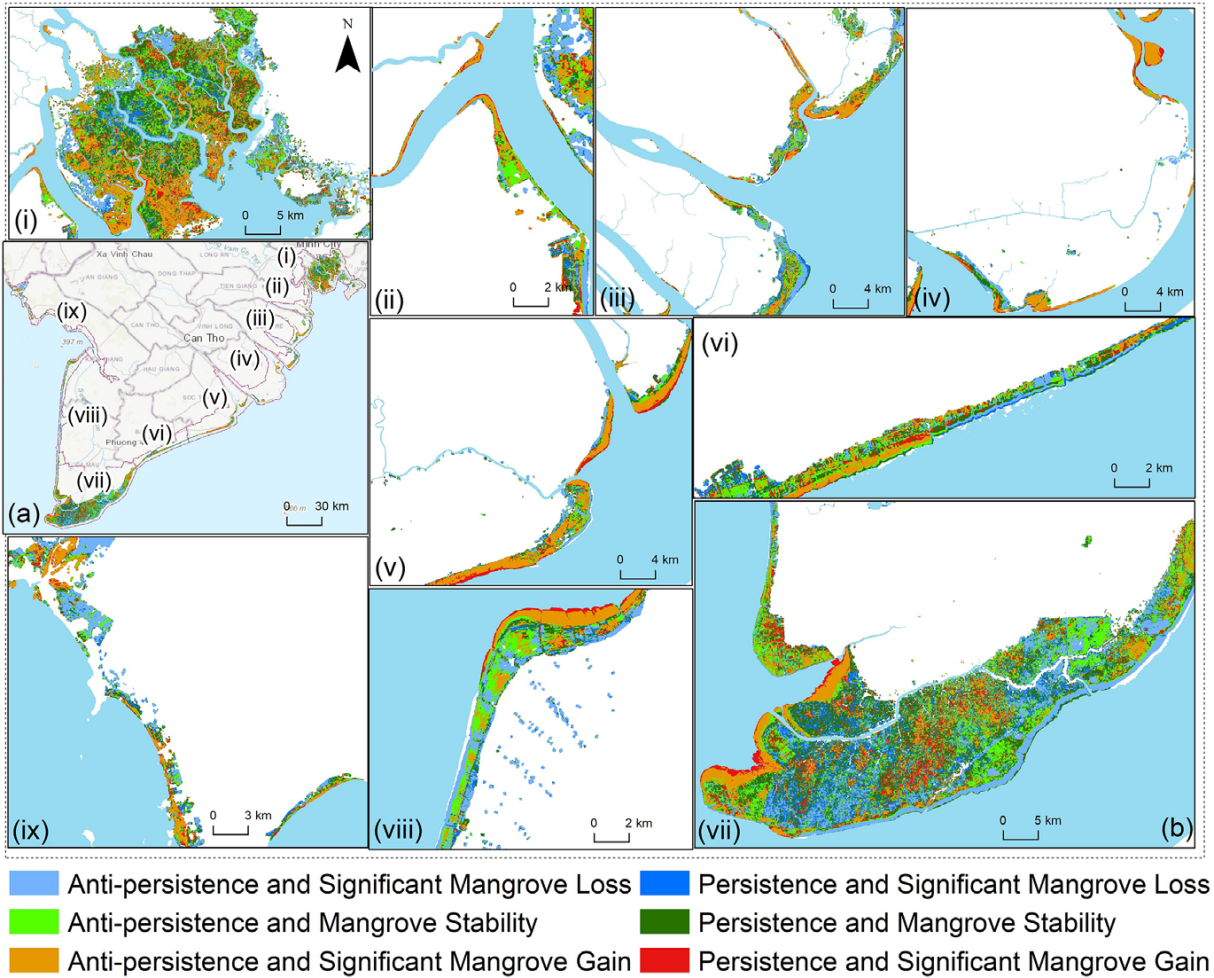
Persistent trends of mangroves-based Landsat NDVI in the study area from 1988 to 2023. The numbered list includes: (i) Can Gio area, (ii) Long An, (iii) Tien Giang, (iv) Ben Tre, (v) Tra Vinh, (vi) Soc Trang and Bac Lieu, (vii) Ca Mau, (viii) Ca Mau West and (ix) Kien Giang.

Sustaining mangrove restoration requires ongoing monitoring and adaptive management. The spatial regression analysis methods applied in the study—such as the Theil-Sen slope, Mann–Kendall test and Hurst exponent—highlight areas experiencing both positive and negative trends over time. Adaptive management allows for periodic reassessment of these trends, ensuring that restoration strategies can be adjusted as conditions evolve. For example, regions like Bac Lieu (Figure 5.vi) and the eastern Ca Mau Peninsula (Figure 5.vii), which showed persistent negative trends, may require tailored or intensified restoration measures compared to more resilient areas like Can Gio (Figure 5.i), where positive trends were more prevalent. Continuous monitoring not only enhances the effectiveness of restoration efforts but also strengthens the resilience of mangrove ecosystems, ensuring their long-term survival amidst the growing pressures of climate change and human intervention.

**Figure 6.**
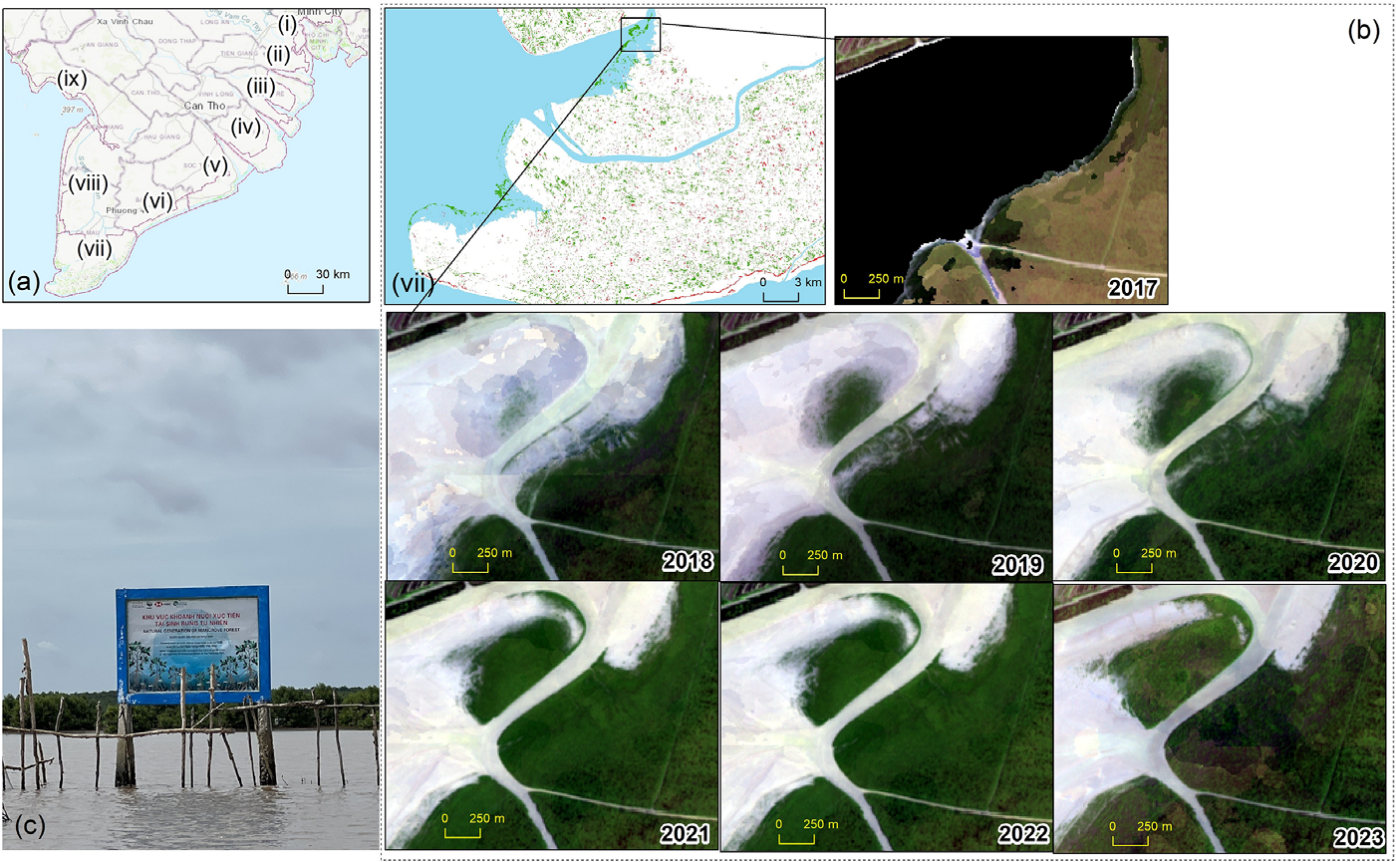
(a) Map displaying the number of regions in the Vietnamese Southern Coastal (VSC) area, with (vii) marking the Ca Mau Peninsula. (b) Location of the islet where natural mangrove regeneration occurred between 2017 and 2023 using composite RGB imagery, obtained from Google Earth Engine. (c) Field photo taken by T.V.T in 2023.

**Figure 7.**
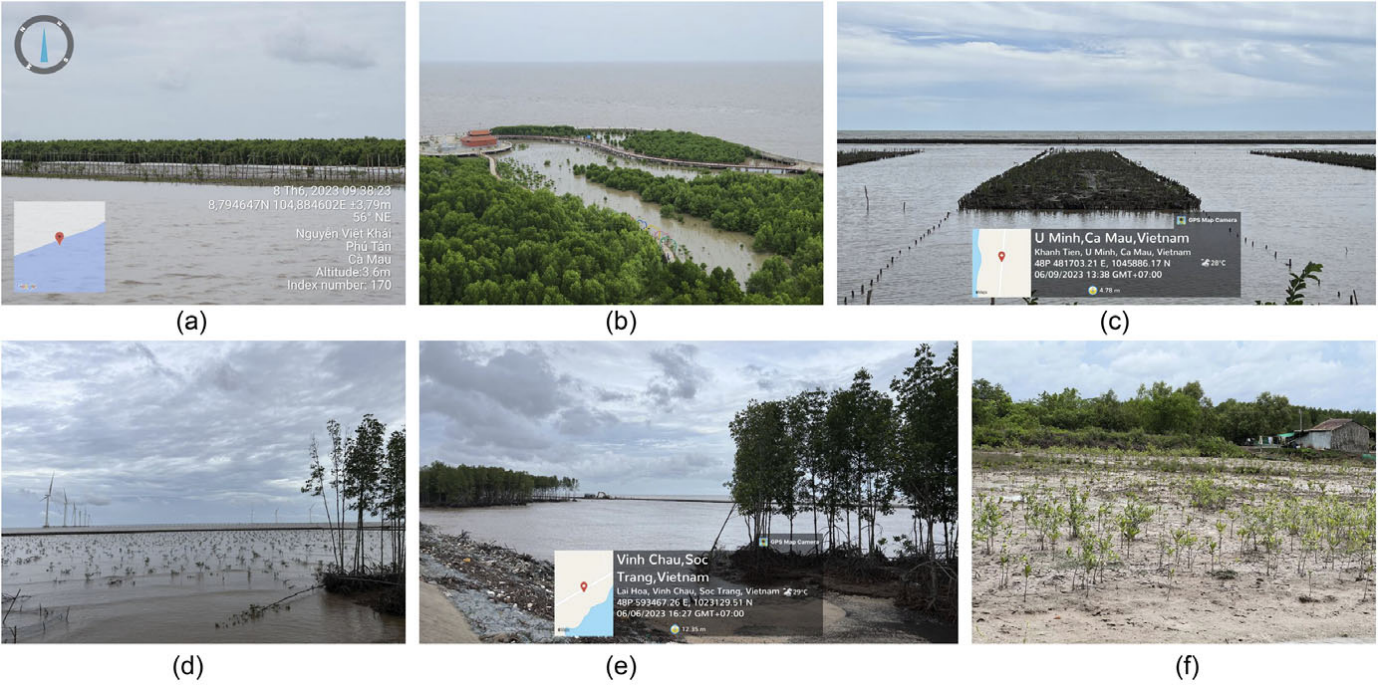
Examples of activities in mangrove restoration include: (a) Groins were installed to trap sediments, creating favourable conditions for mangrove growth on the islet (referenced in Figure 6); (b) planting mangroves in a tourism area; (c) preparation of land for mangrove plantation; (d) planting mangroves following the completion of wind farm construction; (e) a dyke designed to protect mangroves from wave action and (f) planting mangroves in shrimp farming areas. All field photos were taken by T.V.T in 2023.

### Challenges and next steps

The study provided valuable insights into the spatiotemporal dynamics of mangrove ecosystems in the VSC, but it highlighted several limitations that need further exploration at multiple scales. A key challenge is the inherent bias from the short duration of the final dataset segment (2018–2023), potentially skewing long-term restoration interpretations. Frequent cloud cover in tropical regions also complicates NDVI accuracy, suggesting the need for more cloud-free datasets using synthetic aperture radar (SAR) or optical-radar data fusion (Pham et al., 2019). Long-term monitoring is critical to understanding mangrove sustainability. While technologies such as UAVs, LiDAR and SAR can offer detailed insights into biomass, structural changes and carbon sequestration, integrating them with long-term optical datasets is necessary for robust trend analysis. Field validation at focal sites would enhance the accuracy assessments, ensuring that remotely sensed trends align with on-the-ground conditions. By combining multi-source remote sensing data, we can achieve a more comprehensive understanding of mangrove dynamics, bridging the gap between large-scale satellite observations and high-resolution field assessments. Future efforts should focus on developing hybrid approaches that merge optical, radar and in situ datasets to create more robust, scalable models for mangrove restoration monitoring and decision-making. The motives behind restoration—whether for carbon development or sustainable livelihoods—will dictate the appropriate remote sensing techniques and expected results. Additionally, understanding of historical landscape dynamics, even before the Landsat era, is essential for setting realistic restoration goals. Addressing forest fragmentation and integrating environmental variables such as salinity and tidal flow, along with developing secondary forest maps for Southeast Asia, will support more effective restoration strategies and improved ecosystem resilience. These approaches require further study across multiple scales to optimise restoration efforts.

## Conclusion

Mangrove ecosystems are vital for coastal resilience, biodiversity and carbon sequestration, yet they remain highly vulnerable to human activities and natural disturbances. This study offers a detailed assessment of mangrove cover changes in the VSC region from 1988 to 2023, utilising Landsat-based NDVI data and spatial regression techniques to evaluate both degradation and restoration trends. Over the 35-year period, a significant degradation of 68,842 ha (−48.5%) of mangroves was recorded, largely occurring between 1988 and 2018. The eastern area of the Ca Mau Peninsula and Bac Lieu were particularly affected, with the unprotected areas of Ca Mau losing 32,710 ha (76.6%) between 1988 and 1998 alone. However, signs of recovery became evident as 15,033 ha (40.6%) were restored from 1998 to 2008, indicating a shift towards stabilisation.

Restoration efforts have had notable success, with a total gain of 73,117 ha (51.5%) in the mangrove area, particularly in regions like Can Gio and the western Ca Mau Peninsula. Can Gio, in particular, saw a remarkable recovery, gaining 18,938 ha (80.3%) between 1988 and 1998, followed by an even more impressive 90.2% increase by 2023. This positive trend continued with an 83.4% net gain from 2018 to 2023, underscoring the effectiveness of afforestation efforts and community-driven programs. Other regions, such as Bac Lieu and Ben Tre, exhibited mixed trends of both gains and losses, playing secondary roles in the broader restoration narrative. These findings underscore the growing success of restoration initiatives supported by improved governance, hydrological management and enhanced policy frameworks aimed at mangrove conservation.

The methodology applied in this study provides a versatile framework for land change analysis, suitable for different regions, timeframes, satellite data and biomes globally. The results contribute to a comprehensive understanding of mangrove dynamics, which will aid policymakers in crafting effective conservation strategies to support the 11th, 13th and 15th Sustainable Development Goals. While the approach used here offers valuable insights into long-term mangrove change, there is room for improvement in future research. Future studies should consider the influence of sea-level rise and changes in river flow, which could impact mangrove dynamics and provide a clearer basis for long-term projections. Additionally, incorporating seasonal variability, water-use efficiency and mangrove growth rates would further enhance the understanding of these ecosystems and their role in climate resilience and ecological health.

## Data Availability

The data that support the findings of this study are available from the corresponding author upon reasonable request.
