# Supplementary material for: Spectral analysis for monitoring mangrove restoration: A case study in the Vietnamese Southern Coastline
Source: Camb Prism Coast Futur. 2025 Mar 28;3:e8. doi: 10.1017/cft.2025.5 (PMC12337559; doi:10.1017/cft.2025.5)
Supplement: Peer review history

# Reviewer report: Review: Spectral analysis for monitoring mangrove restoration: A case study in the Vietnamese Southern Coastline — R0/PR2

**DOI:** 10.1017/cft.2025.5.pr2

**Authors:** Sarah Hamylton

**Affiliations:** Faculty of Science, Medicine and Health, University of Wollongong, Australia

This study of mangrove change takes place in a key area of global interest in mangrove forest fluctuation. As such, I believe it deserves publication, however, the global insights need to be elaborated to engage a wider audience and take this beyond a site specific study. See manuscript pdf for detailed comments.

# Editor report: Recommendation: Spectral analysis for monitoring mangrove restoration: A case study in the Vietnamese Southern Coastline — R0/PR3

**DOI:** 10.1017/cft.2025.5.pr3

**Affiliations:** Cockrell School of Engineering, The University of Texas at Austin, United States

Thank you for this submission. The manuscript has been reviewed and revisions are minor but important. I look forward to receiving your revised manuscript.

# Editor report: Decision: Spectral analysis for monitoring mangrove restoration: A case study in the Vietnamese Southern Coastline — R0/PR4

**DOI:** 10.1017/cft.2025.5.pr4

**Affiliations:** Geography, Cambridge University, United Kingdom of Great Britain and Northern Ireland

No accompanying comment.

# Editor report: Recommendation: Spectral analysis for monitoring mangrove restoration: A case study in the Vietnamese Southern Coastline — R1/PR6

**DOI:** 10.1017/cft.2025.5.pr6

**Affiliations:** Cockrell School of Engineering, The University of Texas at Austin, United States

thank you for your thorough reviews! I have marked some additional minor edits for the final version:

line 20 - use “delivers” 

line 121 - remove “also”

line 141 - remove “in”

# Editor report: Decision: Spectral analysis for monitoring mangrove restoration: A case study in the Vietnamese Southern Coastline — R1/PR7

**DOI:** 10.1017/cft.2025.5.pr7

**Affiliations:** Geography, Cambridge University, United Kingdom of Great Britain and Northern Ireland

No accompanying comment.
